# Introducing the “urine biochemical approach”: an alternative tool for improving acute kidney injury monitoring in critically ill patients

**DOI:** 10.3389/fneph.2025.1525551

**Published:** 2025-02-19

**Authors:** Alexandre Toledo Maciel

**Affiliations:** ^1^ Head of Research Department, Imed Group, São Paulo, SP, Brazil; ^2^ Adult Intensive Care Unit, São Camilo Pompéia Hospital, São Paulo, SP, Brazil

**Keywords:** acute kidney injury, monitoring, fractional excretion of potassium, urinary sodium concentration, urine biochemical approach

## Abstract

Urine electrolytes and indices assessment as a tool for acute kidney injury (AKI) pathophysiological understanding and management is, until these days, a matter of debate. The classic division of AKI in “pre-renal” (functional/transient) and “renal” (structural/persistent) based on the urinary concentration of sodium and the fractional excretions of sodium and urea has gained popularity for decades and is still present in medical textbooks. Nevertheless, the conclusions of the studies that have used these parameters are very heterogenous and controversial. In the last decade, the pre-renal paradigm has been questioned since urine biochemistry (UB) compatible with “pre-renal AKI” was retrieved from experimental animals with increased renal blood flow, leading some authors to conclude that this approach is not useful for AKI monitoring. Our group has also studied the use of UB in AKI and we think that the key point for adequate use of this tool in clinical practice is a complete mindset change in the way we look and interpret data. In this article, we present the “urine biochemical approach” as an alternative way for UB assessment, which we believe that makes more sense and seems to be more useful for AKI monitoring than the traditional approach. Although the real utility of this alternative approach needs to be confirmed in large, prospective studies, the aim of the present article is to open the mind of critical care practitioners for a potential reappraisal of ancient concepts and ideas regarding the use of urine electrolytes in AKI monitoring.

## Introduction

Acute kidney injury (AKI) is a serious, life-threatening organ dysfunction that carries a poor outcome, particularly among critically ill patients. The increased morbimortality inherent to AKI establishment imposes an urgent search for alternative ways to signal risk or early development of this condition. Although the RIFLE criteria (1) determined that “risk” is when there are subtle increases in serum creatinine (sCr), it is well established that even discrete increases in sCr are a late finding in the process of glomerular filtration rate (GFR) decline. Therefore, an ideal parameter for AKI monitoring should be more sensitive and must point to renal dysfunction when sCr has not yet increased. In this issue, many serum and urinary biomarkers (2) have been proposed to be useful but none of them is fully available in most intensive care units (ICUs), especially in low-income countries, with questionable cost-effectiveness to justify their use in large scale.

On the other hand, our group has described early changes in the urinary electrolyte composition that occur precociously during AKI development, usually one or two days earlier than sCr-based AKI diagnosis (3, 4), leading to what was called the “urine biochemical approach” to AKI monitoring (5). Until now, the two most relevant parameters used in this approach are the urinary sodium concentration (NaU) and the fractional excretion of potassium (FeK).

## The modern interpretation of urine biochemistry in AKI monitoring

### Low NaU and (very) low FeNa: markers of renal microcirculatory stress and increased risk of AKI development

Although widely studied in previous decades, urine biochemistry (UB) evaluation as a tool for AKI monitoring was always controversial and frequently criticized. For many years, a low NaU concentration and a low fractional excretion of sodium (FeNa) were both interpreted as surrogates of low renal perfusion and “pre-renal AKI” (6, 7) a misconception and a broken paradigm in view of the current knowledge of renal physiology and pathophysiology (8, 9). The appealing, didactical separation in functional versus structural AKI based on FeNa and NaU values to determine the best therapeutical approach was progressively put aside (9).

However, the UB abandonment as a tool for renal function assessment seems equally unjustified. In the modern era of UB interpretation, low FeNa and low NaU, instead of representing low renal blood flow (RBF), actually represent renal microcirculatory stress (RMS) with its following activation of sodium-retaining mechanisms (10, 11). Such activation may occur even in the presence of increased RBF as occurs in systemic inflammatory states (sepsis, postoperative, etc) (12). NaU has an advantage over FeNa because it is a result of both glomerular sodium filtration and tubular sodium reabsorption, while FeNa is a parameter of tubular reabsorption only. Yet, FeNa is already low (around 0.5%) in critically ill patients with preserved renal function (3) so that, in the presence of RMS or declining GFR, the additional decrease in FeNa value is limited. In other words, FeNa has no direct relation with GFR. In the presence of a low GFR, both a high or a low FeNa may be observed, depending on the integrity of tubular cells and their capability to reabsorb sodium. Nonetheless, a low GFR is always followed by a decreased NaU, with the magnitude of this decrease depending on the integrity of tubular cells. Conversely, a high NaU, defined as a concentration higher than its equivalent in serum (13), is theoretically only possible in the presence of high GFR and, consequently, high sodium filtration. If low sodium is filtered, even with jeopardized tubular sodium reabsorption, the augmentation in NaU is limited. In the early phases of AKI development, significant and acute decreases in NaU occur (3), reflecting reduced sodium filtration and activated sodium reabsorption. Renal recovery is usually followed by relevant increases in natriuresis in contrast to limited increases in NaU that may occur with progressive tubular damage (acute tubular necrosis), especially considering simultaneous sodium back-leak occurrence.

Interestingly, natriuresis has a tight correlation with inflammation. Systemic inflammatory states are characterized by avid-sodium retention and it is not infrequent that natriuresis recovery occurs later than decreases in sCr and urinary flow improvement (14). Diuresis recovery without simultaneous natriuresis recovery may lead to a *diabetes insipidus*-like behavior leading to hypernatremia, as we have frequently observed in our ICU. A low NaU is usually found in the presence of high C-reactive protein values and usually parallels with high serum urea (sUr) values because avid sodium retention occurs in great proportion at the proximal tubule, where most of the filtered urea is also being avidly reabsorbed.

## Is there a role for the fractional excretion of urea in the urine biochemical approach?

The fractional excretion of urea (FeUr) has been used for a long time as a surrogate of FeNa to discriminate between transient (tAKI) and persistent AKI (pAKI) particularly when diuretics were recently administered. The classic cutoff value is 35%. The theoretical advantage of FeUr in this situation is that its value is less influenced by diuretic use since most filtered urea is reabsorbed at the proximal tubules, preventing the influence of diuretics which usually act more distally in the nephron. However, there is a lot of controversy and conflicting results regarding the capability of both FeNa and FeUr to be useful in practice to distinguish a “pre-renal” from an intrinsic AKI at the day of AKI diagnosis (15). In a previous study by our group, there were no significant differences in FeUr between tAKI and pAKI at the day of AKI diagnosis (3). Nonetheless, in the urine biochemical approach, all fractional excretions are monitored before AKI diagnosis. In that same study by our group (3), no clear evidence was found that the FeUr behavior is different between tAKI and pAKI considering 2 days before AKI diagnosis as it seems to be the case for FeK (4). This was the reason why FeK was selected as the fractional excretion of choice to compose the urine biochemical approach.

## FeK as a dynamic surrogate of serum creatinine

Similar to NaU, FeK is also a valuable monitoring tool as long as it is being monitored before increases in sCr (4). Considering the 3 most studied fractional excretion parameters (FeNa, FeUr and FeK), the latter is certainly the less studied but, perhaps, the most relevant. This is because the urinary potassium to creatinine concentration (KU/CrU) ratio included in its formula is a valuable marker of the adequacy between urine output (UO) and CrU (10). Additionally, in the normal range of sCr, decreases in GFR are followed by increases in FeK, collaborating to prevent life-threatening increases in serum potassium (16). This is because increases in FeK in the initial process of decreasing GFR helps to minimize the decrease in urinary K^+^ excretion. K^+^ is secreted by distal tubules in exchange for Na^+^, which is being avidly reabsorbed during RMS situations, as previously mentioned. Some authors have proposed to monitor decreases in urinary K^+^ excretion using 2-h urine sample as an early sign of renal impairment (17, 18). Since increases in FeK delays the decrease in urinary K^+^ excretion, it is expected that increases in FeK will occur earlier and, consequently, be an even more precocious sign of decreasing GFR.

It is not possible to monitor renal function in a timely manner without including urinary parameters. Notably, the single urinary parameter measured in current clinical practice is UO but this is not enough for an early AKI diagnosis. Urine composition may be even more relevant than its volume. Many urinary biomarkers have been demonstrated to be precociously increased during AKI development but, unfortunately, most are currently not available in most centers, besides the fact that they are usually not affordable. On the other hand, the urine electrolyte composition seems to be helpful without significant additional costs, which favors its widespread and repeated use. In situations with potential abrupt decreases in renal function, FeK monitoring may be quite more useful than only sCr and UO (19). This is because sCr refers to the renal function at a recent past and decreases in UO are not mandatorily dysfunctional, especially in surgical patients (20). The presence of a concentrated urine at the collecting bag usually motivates the prescription of more fluids with the idea that a decreased urine volume is a specific marker of hypovolemia which is usually not the case, especially if its composition suggests a benign, physiological oliguria. At this point, FeK evaluation is relevant since normal FeK (below 10-12%) in oliguric patients probably signals an adapted creatinine excretion in a smaller urine volume: no significant increases in sCr are expected in the subsequent hours (10) (see below).

## Urine biochemistry as the missing gap between serum creatinine and urine output

The limitations of sCr and UO as markers of AKI are well established. Even hypothetically considering a decreasing creatinine clearance (CrCl) as a gold standard for GFR impairment and AKI diagnosis, it is hard to assess CrCl repeatedly in a practical way at bedside. For instance, a patient with normal sCr does not necessarily need to have significant decreases in UO to alert for AKI development. The opposite is also true: AKI recovery occurs before sCr begin to decrease and increasing urine volume is not mandatorily present. In fact, it is difficult to predict on-time behavior of creatinine excretion based on a static sCr measurement and some hours of UO assessment in situations of potentially rapid changes in GFR. As previously mentioned, the effect of the UO on sCr essentially depends on urine composition (21), which is usually the first element that will signal both AKI development and recovery.

## Focusing on urine composition instead of serum creatinine and urine output

In the urine biochemical approach, increases in sCr are interpreted as the last step of the sequential chain of pathological events related to AKI development (5). As previously demonstrated, decreases in NaU and increases in FeK occur 1-2 days earlier than sCr-based AKI diagnosis (3). In addition, distinction between sCr-based and UO-based AKI diagnosis as preconized in RIFLE, AKIN and KDIGO criteria lack the theoretical relevance that urine composition may have to define prognosis. It remains to be established if the prognostic impact of oliguria is independent of urine composition. It is possible that FeK and NaU may be determinants of outcome considering similar levels of UO and sCr but additional studies are needed to confirm this hypothesis.

## The very early phase of AKI development: stressed vs dysfunctional kidneys

Many clinical conditions may lead to avid-sodium retention which is a hallmark of RMS. However, it is important to distinguish RMS from dysfunctional kidneys. Although RMS is a very relevant risk factor for renal dysfunction (and this is the most important reason why it must be monitored and diagnosed), stressed kidneys may remain functional with a preserved capability to excrete creatinine properly. In order to distinguish functional and dysfunctional stressed kidneys while using the urine biochemical approach, low NaU values might be accompanied by normal and increased FeK values, respectively. In all cases, sUr and sCr are considered to still be at normal values, insuring an early stage of monitoring. AKI is then a posterior stage due to persistent dysfunctional kidneys. As previously mentioned, the proposal of urine biochemical approach is to establish that sUr and sCr are both the result of a *recent* renal function but not *current* renal function. In this issue, an increasing FeK signals dysfunctional kidneys (decreasing GFR) at the present moment, leading to potential accumulation of nitrogenous waste products and AKI diagnosis in the near future. Nevertheless, it is possible for stressed kidneys to remain functional with normal FeK values, indicating adequate creatinine excretion, regardless of in a high or low urine volume (the so called “permissive oliguria”) (20).

## Classifying renal function before AKI diagnosis: proposed terminologies

There are a lot of possible renal conditions behind a normal sUr and sCr values (**Table 1**). The first insight while facing these normal values is to understand that they do not represent normal renal function in a timely manner. Albeit usually increasing and decreasing together, sUr and sCr may behave differently one from the other, particularly in systemic inflammatory states such as sepsis, trauma and postoperative. The avid sodium retention that characterizes systemic inflammation is simultaneously followed by avid tubular urea retention, so that sUr levels tend to be proportionally higher in comparison to sCr. Contrary to previous paradigm that a high sUr/sCr ratio is a hallmark of low renal perfusion states (22), in fact it is characteristic of RMS, once excluded non-renal causes of an elevated ratio such as gastrointestinal bleeding, corticosteroids use, elevated dietary protein intake or low sCr due to malnutrition. Stressed kidneys may excrete creatinine properly while avidly reabsorbing urea. During AKI recovery, sCr may decrease faster than sUr, meaning that GFR recovery may occur earlier than attenuation of the avid tubular sodium and urea retention. In order to classify renal function *before* increases in sCr and independently of UO (because decreases in UO are not mandatorily pathologic), we have proposed the following terms:

**Table 1:** Differences in the traditional versus urine biochemical approach for AKI monitoring.

Traditional approach		Urine biochemical approach	
Normal sUr and sCr and No previous information regarding UO			
Renal function considered to be normal at this moment		Renal function considered to be normal in a recent past (some hours ago) -Is renal function still normal at this moment?	
Follow UO for 6 hours + sUr and sCr assessment in the next routine lab		Spot urine sample assessment – measure NaU and calculate FeK Low NaU (< 40) – RMS (stressed kidneys)- increased AKI risk High FeK (> 12) – dysfunctional kidneys- increases in sCr expected Alert sign – renal function is NOT normal at this moment- check early for possible reversible causes of renal impairment; avoid additional insult	
Decreasing UO (< 0.5 ml/kg/h)	Normal UO (≥ 0.5 ml/kg/h)	Follow UO + urine electrolyte composition for 6 hours	
AKI development (UO criteria) Increased sCr expected in the next measurement	Renal function remains normal	Decreasing UO + increased FeK- pathologic oliguria Increased sCr expected in the next measurement AKI development	Decreasing UO with normal KU/CrU ratio (normal FeK) – permissive/physiological oliguria (better prognosis than UO AKI criteria?) – no expected increases in sCr No AKI development
New sUr and sCr assessment 6 hours after initial measurement (possible sCr AKI criteria)	New sUr and sCr assessment in the routine lab Normal values expected	Low NaU with normal FeK: stressed but functional kidneys Increased sUr/sCr ratio (isolated elevation in sUr with normal sCr)	Normal or increased UO with increased FeK (low KU and disproportionately low CrU resulting in high KU/CrU ratio) Expected increases in sCr non-oliguric sCr-based AKI development

AKI: acute kidney injury; sUr: serum urea; sCr: serum creatinine; UO: urine output; NaU: urinary sodium concentration; FeK: fractional excretion of potassium; RMS: renal microcirculatory stress; KU/CrU: urinary potassium (mEq/L)/urinary creatinine concentration (mg/dL). "normal FeK value: below 10-12%" and "normal KU/CrU ratio: around 0.5 but can be physiologically higher in cases of very low sCr".

Stressed kidneys

- avid sodium and urea retention by the tubules, decreasing NaU, FeNa and FeUr
- may have normal GFR.
- high sUr/sCr ratio usually present.
- risk factor for dysfunctional kidneys and AKI.

Dysfunctional kidneys

- disproportional decrease in CrU in relation to UO (high KU/CrU ratio), leading to a decreased renal creatinine excretion.
- decreasing GFR expressed by increasing FeK.
- stage that precedes AKI and may revert before AKI establishment (absence or increases in sCr < 0.3 mg/dL in 48 hours).

Permissive oliguria

- usually high KU (low UO) and proportionally high CrU so that KU/CrU ratio remains at normal levels (normal FeK despite low UO). In other words, preserved creatinine excretion despite low UO.

A hypothetical example of the use of both traditional and urine biochemical approach showing different perceptions of the actual renal function is shown in **Table 2**.

**Table 2:** A hypothetical postoperative example of AKI monitoring showing the asynchronous perception of renal impairment and recovery between traditional and urine biochemical approach.

ICU admission	6 hours later	12 hours later	24 hours later	48 hours later
sUr 30 sCr 0.8 sUr/sCr 37.5	sUr 45 sCr 0.9 sUr/sCr 50.0	sUr 70 sCr 1.2 sUr/sCr 58.3	sUr 70 sCr 1.1 sUr/sCr 63.6	sUr 40 sCr 0.8 sUr/sCr 50.0
Intra-op UO: 0.5 ml/kg/h	6 h UO: 0.5 ml/kg/h	12 h UO: 0.4 ml/kg/h	24 h UO: 0.5 ml/kg/h	24 h UO: 0.6 ml/kg/h
NaU 50 FeK 16.0% KU 40 CrU 50 KU/CrU 0.80	NaU 20 FeK 18.4% KU 45 CrU 55 KU/CrU 0.82	NaU 15 FeK 15.0% KU 60 CrU 120 KU/CrU 0.50	NaU 13 FeK 9.8% KU 50 CrU 140 KU/CrU 0.36	NaU 200 FeK 10% KU 30 CrU 60 KU/CrU 0.50
Traditional approach - normal renal function	Traditional approach - normal renal function	Traditional approach - AKI KDIGO 1 - pre-renal AKI	Traditional approach - AKI KDIGO 1 - pre-renal AKI	Traditional approach - AKI resolution
UB approach -dysfunctional kidneys	UB approach -stressed kidneys (RMS) -dysfunctional kidneys (decreasing GFR) - high risk of AKI	UB approach -stressed kidneys (RMS) -recovering GFR	UB approach -stressed kidneys (RMS) -normal GFR	UB approach -RMS resolution -normal GFR

AKI: acute kidney injury; ICU: intensive care unit; sUr: serum urea (mg/dL); sCr: serum creatinine (mg/dL); UO: urine output; NaU: urinary sodium concentration (mEq/L); FeK: fractional excretion of potassium; RMS: renal microcirculatory stress; KU/CrU: urinary potassium (mEq/L)/urinary creatinine concentration (mg/dL); GFR: glomerular filtration rate; UB: urine biochemical. KDIGO: Kidney Disease: Improving Global Outcomes.

Serum K^+^ was considered to be 4 mEq/L in all measurements for FeK calculation.

## Possible misconceptions in previous studies evaluating urinary electrolytes in AKI

**Figure 1:**
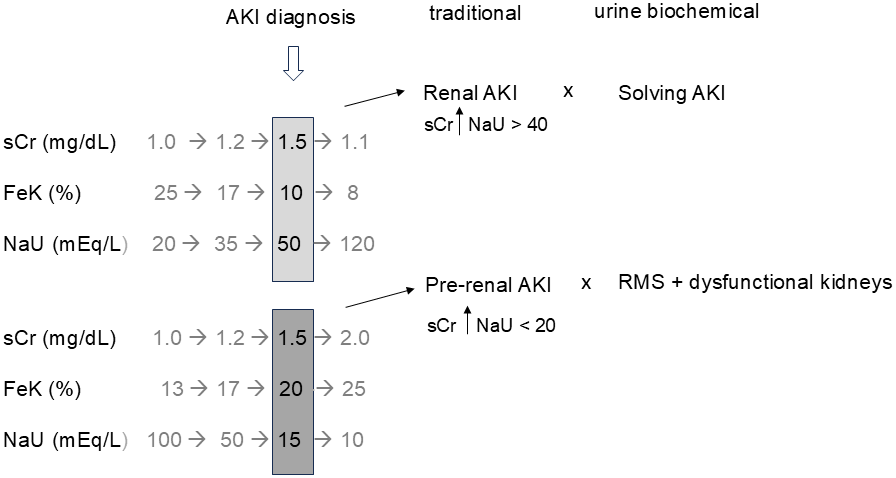
Temporal dissociation between serum creatinine (sCr) and urine biochemical parameters (FeK and NaU) regarding the moment of renal dysfunction diagnosis and recovery. Simultaneous evaluation may lead to random combinations and erroneous interpretations such as “renal (structural) AKI” when renal function is actually recovering and “pre-renal AKI” with no obvious evidence of low renal blood flow in many cases. Renal microcirculatory stress (RMS) is a wider diagnosis which includes any condition that triggers avid tubular sodium reabsorption. Gray numbers represent the “missing story” when a single assessment of sCr and urine biochemistry is made (black numbers), as is common in most studies. AKI, acute kidney injury; FeK, fractional excretion of potassium; NaU, urinary sodium concentration.

There are several reasons why previous studies evaluating urinary electrolytes in AKI have failed to show consistent results that motivated UB widespread use in clinical practice. The main reasons are described below:

1. Avid sodium retention as a synonym of low RBF - the “pre-renal paradigm”
  In the urine biochemical approach, activation of sodium-retaining mechanisms is seen as a sign of RMS, which can be induced by many situations, being low RBF due to hypovolemia or low cardiac output just one of the possibilities that must be excluded, especially because it is one of the possible actively reversible causes. A “microcirculatory view” emphasizes that RMS may occur in the absence of macrocirculatory derangement (12).
2. Urine electrolytes assessment at or after AKI diagnosis
  Notably, the urine biochemical approach proposes renal function monitoring while sUr and sCr are in theoretically normal values. In the absence of reference values, it is hard to know, for instance, if a sCr of 1.1 mg/dL is normal or represent an AKI KDIGO 1. The presence of a high FeK simultaneously with a sCr of 1.1 mg/dL points towards dysfunctional kidneys. In this case, additional increases in sCr might be expected or it might suggest that 1.1 mg/dL already represents an increased sCr value for that patient. In fact, sCr and urinary electrolytes do not represent the same moment of the renal function because sCr takes a longer time to change in response to a change in GFR. That being said, the interpretation of data is frequently random and erroneous when a single-point assessment is made combining simultaneous sCr and urine electrolytes, particularly when sCr is already compatible with AKI, as previously explained in another paper (23). The single-point combination of sCr and NaU, for example, is possibly responsible for many drawbacks in the current use of NaU and other urinary indices to distinguish “pre-renal” and “renal” AKI (**Figure 1**).
3. Neglected FeK as the most valuable fractional excretion parameter for AKI monitoring
  In the urine biochemical approach, FeK is a very relevant parameter although it has been neglected for decades. Most studies focused in FeNa and FeUr which do not give the same level of information as FeK, even if they are measured before AKI diagnosis, as is preconized by this approach.

## Limitations of the urine biochemical approach in AKI monitoring

The main limitation of using urine biochemical approach is simultaneous diuretic administration. Diuretics are the class of medications with the greatest potential to modify urine electrolyte composition, jeopardizing the natural behavior of NaU and FeK, artificially increasing both (24). In practical terms, these two variables must be assessed preferably at least 6 hours after the last diuretic administration (25). Other medications such as angiotensin receptor blockers and anti-inflammatories may also interfere in urine electrolyte composition although, in my daily ICU practice, such interference is much lower than that of diuretics and usually do not preclude urine electrolytes assessment. The type and volume of crystalloids being infused may also have a theoretical impact in urine electrolyte composition, particularly in NaU, despite the fact that its sequential behavior (which is more relevant than the absolute value of NaU *per se*) is not usually significantly affected. An exception would be a possible improvement in renal hemodynamics and, consequently, in NaU due to fluid challenge in hypovolemic states. In this case, increases in NaU are a sign of renal improvement and the type of crystalloid and its composition is probably of less importance.

The fact that a urine sample is needed is also a potential practical limitation except when the patient has an indwelling urinary catheter. Other limitations are (1) chronic renal disease, in which previous glomerular and/or tubular impairment may modify NaU and FeK behaviors; (2) severe AKI, for the same reason. In fact, in my experience, the utility of the urine biochemical approach in AKI recovery if the AKI event was severe (KDIGO 3) is less straightforward; and (3) creatinine production, which is a relevant variable not measured in daily ICU practice that may interfere in all parameters that use both sCr as well as CrU as surrogates of renal function.

## In terms of prognosis, is it relevant to diagnose renal microcirculatory stress or dysfunctional kidneys?

The most important question that remains is whether identifying these subclinical, “pre-AKI” stages will some way open a therapeutic window that may change the course of AKI development and, consequently, patient´s outcome. As an example, should we avoid giving iodinated intravenous contrast (or any other potential nephrotoxic medication) for a patient that have normal sCr but diagnosed with RMS or dysfunctional kidneys? Does the presence of a “pre-AKI” stage imposes an unfavorable evolution or only more significant decreases in renal function such as AKI KDIGO 1 are relevant? All these questions must be answered in future studies but the primary objective of this article is to propose a new, more sensitive approach that may improve AKI monitoring especially in scenarios where acute alterations in renal function are expected.

## Conclusions

Several misinterpretations and conflicting results along the decades have led the use of urine electrolytes to be banished from the armamentarium for AKI monitoring. Our group is proposing a change of mindset regarding this subject with a different view and approach that, in our opinion, make a lot of sense and open a promising line of research that may help early detection of renal impairment (and, eventually, recovery too) without significantly increasing the costs of the critically ill care. In order to confirm the utility of this approach and better define its limitations, new studies from others groups in different scenarios using this alternative perspective are certainly needed.

## Data Availability

The original contributions presented in the study are included in the article/supplementary material. Further inquiries can be directed to the corresponding author.
